# Abnormalities of saccadic eye movements in dementia due to Alzheimer’s disease and mild cognitive impairment

**DOI:** 10.18632/aging.102118

**Published:** 2019-08-02

**Authors:** Thomas D.W. Wilcockson, Diako Mardanbegi, Baiqiang Xia, Simon Taylor, Pete Sawyer, Hans W. Gellersen, Ira Leroi, Rebecca Killick, Trevor J. Crawford

**Affiliations:** 1 School of Sport, Exercise and Health Sciences, Loughborough University, Loughborough, UK; 2 Psychology Department, Lancaster University, Lancaster, UK; 3 Computing and Communications Department, Lancaster University, Lancaster, UK; 4 Faculty of Information Technology and Electrical Engineering, University of Oulu, Oulu, Finland; 5 Maths and Statistics Department, Lancaster University, Lancaster, UK; 6 Engineering & Applied Science, Aston University, Birmingham, UK; 7 Global Brain Health Institute, Trinity College, Dublin, Ireland; 8 Division of Neuroscience and Experimental Psychology, University of Manchester, Manchester, UK; 9 Greater Manchester Mental Health NHS Foundation Trust, Manchester, UK

**Keywords:** mild cognitive impairment, Alzheimer’s disease, inhibitory control, eye tracking, anti-saccade

## Abstract

Background*:* There is increasing evidence that people in the early stages of Alzheimer’s disease (AD) have subtle impairments in cognitive inhibition that can be detected by using relatively simple eye-tracking paradigms, but these subtle impairments are often missed by traditional cognitive assessments. People with mild cognitive impairment (MCI) are at an increased likelihood of dementia due to AD. No study has yet investigated and contrasted the MCI subtypes in relation to eye movement performance. Methods*:* In this work we explore whether eye-tracking impairments can distinguish between patients with the amnesic and the non-amnesic variants of MCI. Participants were 68 people with dementia due to AD, 42 had a diagnosis of aMCI, and 47 had a diagnosis of naMCI, and 92 age-matched cognitively healthy controls. Results: The findings revealed that eye-tracking can distinguish between the two forms of MCI. Conclusions*:* The work provides further support for eye-tracking as a useful diagnostic biomarker in the assessment of dementia.

## Introduction

Alzheimer's disease (AD) is a severe neurodegenerative disease of the human brain, for which there is as yet no cure. When a disease modifying therapy becomes available, it will be essential to administer this treatment in the very earliest stages of the disease, before pathological changes in the brain are widespread, rendering the treatment ineffective [1]. Thus, identifying the presence of AD in the pre-dementia ‘prodromal’ or even ‘preclinical’ phase is essential [2]. Current biomarkers that are able to detect AD in the earliest stages are either invasive (i.e. involving a lumbar puncture to a cerebrospinal fluid sample) or expensive (i.e. involving neuroimaging). Thus, the discovery of a reliable non-invasive and low-cost biomarker would be an important development with implications for the early diagnosis and monitoring of the disease, particularly on a global scale, since invasive and/or expensive biomarkers may not be widely available.

Eye movements provide a sensitive, low cost and non-invasive marker of cognitive change or deterioration [3,4]. People with AD gradually lose the efficient control of attention and develop impairments of both inhibitory control and eye movement error-correction [5]. Specifically, the inhibition of a gaze-shift towards a salient stimulus as well as the ability to direct a voluntary gaze-shift away from this stimulus is impaired. This difficulty in gaze control may be due to cognitive defects of either inhibitory control, working memory (WM), or both [6,7]. The error frequency in the antisaccade task (AST) also correlates with the severity of AD [3]. Importantly, eye movement deficits can develop early in the course of the disease, even before cognitive deficits are revealed by standard neuropsychological tests [3,7]. A critical issue then is whether eye-movement impairments are detectable in people who are in a preclinical stage of AD and therefore at a greater risk of developing clinical dementia.

A strong correlation has been reported [8] between antisaccade error rate with cortical thinning (brain atrophy) in a mild cognitive impairment (MCI) group. However, this work did not distinguish between the different types of MCI, thus the low and high-risk of dementia participants were conflated in their study. In a separate study, a group of amnesic MCI (aMCI) participants were contrasted to age-matched controls who were assessed both using the AST and with fMRI to measure structural changes [9]. The fMRI data revealed that participants with aMCI showed reduced activation in frontal eye fields and increased inhibitory errors when performing the AST. Although this work supports eye-tracking as a useful diagnostic tool in detecting individuals at high risk of dementia, the evidence was not conclusive, as there was no direct comparison between an aMCI and a non-amnesic MCI (naMCI) group. Evidence that saccadic impairment was significantly greater in the aMCI group with the higher risk for dementia due to AD, in comparison to the naMCI group who are at a lower risk, would clearly provide more compelling support for the validity of the AST as an early diagnostic marker.

A clinically useful biomarker should be able to detect subtle signs of impairment in a group of participants at higher risk of dementia such as those with MCI. MCI is a clinical syndrome characterised by impairments in cognition that are worse than would be expected for a person of their age. MCI has several outcomes; it can lead to dementia if the underlying cause is AD, or other types of neurodegenerative conditions; or it may be transient, reversible [10] or even static. People with a diagnosis of MCI are at an increased risk of developing dementia compared to cognitively healthy adults with 5-10% of MCI patients progressing to dementia annually (see [11]). Traditionally, the clinical syndrome of MCI was considered to be a relatively distinct stage of dementia since the cognitive deficits were not severe enough to impact significantly on the individual’s ability to conduct their activities of daily living [12]. However, there is a growing consensus [13,1,2] that MCI should be considered a prodromal and/or preclinical stage between normal cognitive health and AD. MCI can be further split into aMCI and naMCI types [12]. Those with aMCI experience greater memory impairment in comparison to naMCI. Further [14], found evidence that there are structural differences between aMCI and naMCI in certain brain regions (the largest differences found for the hippocampus and entorhinal cortex). This suggests that memory differences may be the result of physical changes in the brain. Critically, there is a higher conversation rate to dementia due to AD for people diagnosed with aMCI, than naMCI [15–17]. Thus, people with aMCI are at much greater risk of progressing to AD than healthy adults or naMCI. No study has yet investigated the comparison of MCI subtypes in relation to eye movement performance. The principal aim of the current work was to explore and evaluate such evidence.

## RESULTS

### Antisaccade latency

**Figure 1:**
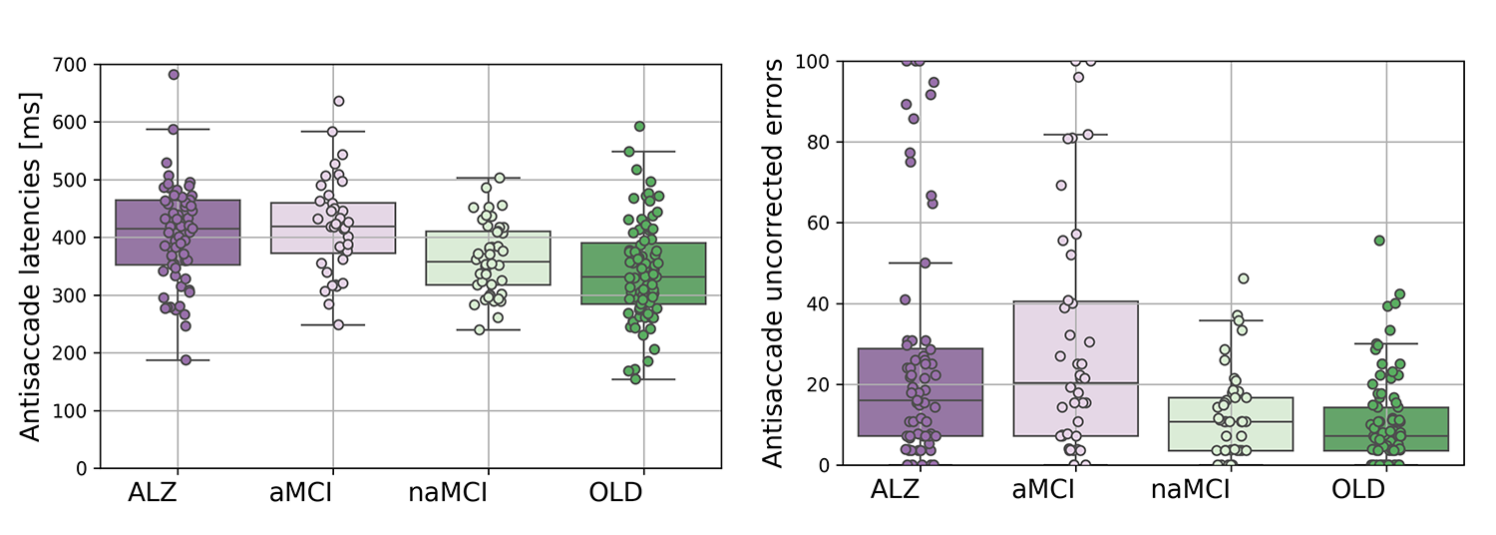
**The eye movement variables for the Alzheimer's disease (AD), amnesic Mild Cognitive Impairment (aMCI), non-amnesic Mild Cognitive Impairment (naMCI), and control participants (CP).** (**A**) Antisaccade latencies (left panel); (**B**) Antisaccade uncorrected errors (right panel).

The latencies for all correct saccades in the antisaccade are shown in Figure 1A. A one-way ANOVA on the mean antisaccade latencies revealed that there was a significant main effect of participant group (F(3,238)=13.541;p<.005; η^2=^.146). AD (N=65; mean=404; SD=86; 95% CI=383-427) generated significantly longer latencies than the CP group (N=91; mean=338ms; SD=84; 95% CI=321 -355; t(154)=4.801;p<.0005; *d*=.77) and the naMCIs (N=46; mean=363ms; SD=62; 95% CI=346-381; t(109)=2.78;p=.006; *d*=.53). However, antisaccade latencies did not differ between the aMCI (N=40; mean=419ms; SD=82; 95% CI=394-444) and the AD group (t(103)=.857;p=.394; *d*=.17). The mean latencies of aMCI group were significantly longer than the naMCI group (t(84)=3.607;p=.001; *d*=.79), and the CP group (t(129)=5.116;p<.0005; *d*=.90). The latencies for the naMCI did not differ from the CP group significantly (t(135)=1.785;p=.077; *d*=.31). Because age was not balanced between the groups we next performed an analysis of covariance. The ANCOVA, with a between-subjects factor: group (AD, aMCI, naMCI, CP); and a covariate: age, revealed a significant main effect of age (F(1,230)=32.115;p<.0005; η^2=^.123), and group (F(3,230)=6.175;p<.0005; η^2=^.075). However, the corrected model, with age as a covariate was also significant (F(4,230)=18.087;p<.0005; η^2=^.239), confirming that the group effect remained after age was partialled out. To provide further verification that age did not affect results we selected a subsample of age-matched participants. This age-matched sample was achieved by excluding the oldest AD (N=4) and aMCI (N=3) and the youngest CPs (N=22) and naMCI (N=9) participants from the sample. An ANOVA confirmed that the groups were matched across age (F(3,201)=.800;p=.495; η^2=^.012). The antisaccade latency findings were replicated in this age-matched subsample. ANOVA revealed a significant main effect of participant group for mean antisaccade latencies (F(3,201)=7.263;p<.0005; η^2=^.098). AD (N=62; mean=402ms; SD=88; 95% CI=381-425) revealed increased latencies in comparison to the CP group (N=69; mean=351ms; SD=88; 95% CI=331-374; t(129)=3.304;p=.001; *d*=.58), and the naMCI group (N=37; mean=363; SD=64; 95% CI=343-383; t(97)=2.311;p=.023; *d*=.47). Again, the aMCI (N=37; mean=418; SD=83; 95% CI=392-445) did not differ from AD (t(97)=.910;p=.365; *d*=.19). The aMCI group revealed significantly longer latencies compared to the naMCI group (t(72)=3.160;p=.002; *d*=.75), and the CP group (t(104)=3.811;p<.0005; *d*=.75). The antisaccade latencies for the naMCI group did not differ from CP group (t(104)=.759;p=.449; *d*=.15).

### Antisaccade uncorrected errors

**Figure 2:**
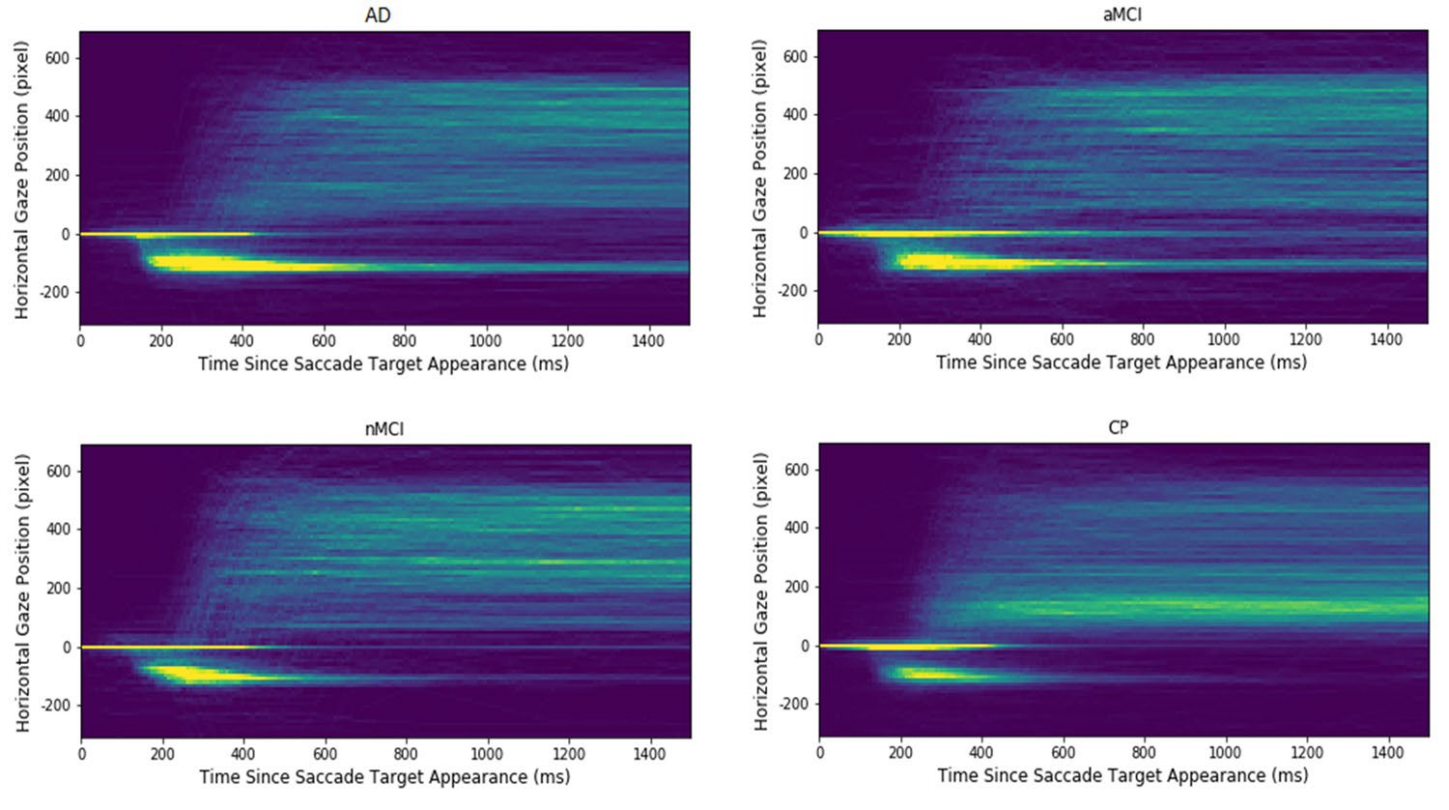
**Heatmap plots of the extracted gaze signals in each of participant groups.** The x-axis indicates the time since saccadic target appearance, and the y-axis presents the aligned horizontal gaze position. The warmer the colour; the higher is the gaze point density in the corresponding spatial-temporal location. Note that the longest "comet" tails, reflecting a high proportion of uncorrected errors, are evident for the Alzheimer's (AD) and the amnesic Mild Cognitive Impairment (aMCI) groups. The control participants (CP) and the non- amnesic MCI have distinctly shorter "comet" tails.

Figure 2B shows the mean frequency (%) total number of trials with uncorrected errors in each group for the antisaccade experiment. A logistic regression [18] was fitted to the proportion of uncorrected errors, with weights proportional to the number of AST completed per participant and a dispersion parameter to account for potential higher than anticipated variation in the number of uncorrected errors. Estimated log-odds regression coefficients, presented in Table 1, identify that the proportion of uncorrected errors are significantly lower than 50% for all groups, and thereby revealing that there was a significant effect on participant group for the antisaccade errors (F(3,245)=10.914; p<.0005). The distribution of the standardised Pearson residuals did not visually appear to satisfy the assumption of the normal distribution. However, a one-way ANOVA test suggest that the residual contain no further dependence on group (F(3,245)=.108; p=.956) and likewise from a non-parametric analysis using the Kruskal Wallis H Test (H(3)=3.073; p=.381).

**Table 1:** Log-odds coefficient estimates for cognitive groups from the weighted logistic regression model for the proportion of uncorrected AST errors.

Group	Log-odds	SE	95% Confidence Interval		Wald
			Lower	Upper	Chi-Square
All participants (N=249), dispersion = 5.361					
AD	-1.242	0.1389	-1.515	-0.970	79.973
aMCI	-1.063	0.1699	-1.396	-0.730	39.192
naMCI	-1.974	0.2038	-2.373	-1.574	93.782
CP	-2.176	0.1670	-2.503	-1.848	169.688
Age matched (N=211), dispersion = 5.645					
AD	-1.226	0.1453	-1.511	-0.941	71.218
aMCI	-1.107	0.1827	-1.466	-0.749	36.742
naMCI	-1.959	0.2317	-2.413	-1.505	71.487
CP	-2.266	0.2066	-2.371	-1.861	120.267

All chi-squared Wald statistics are based on 1 degree of freedom and all correspond to p-values less than 0.0005.

As expected, AD (N=68; mean proportion=26; SD=29; 95% CI=19-33) generated increased errors in comparison to the CP group (N=91; mean proportion=10; SD=11; 95% CI=8-13; χ2(1)=18.459; p<.0005). Critically, the aMCI group (N=42; mean proportion=30; SD=30; 95% CI=21-39) generated a higher proportion of antisaccade errors compared to the naMCI group (N=46; mean proportion=12; SD=11; 95% CI=9-16; χ2(1)=11.774; p=.001) and the CP group (χ2(1)=21.806; p<.0005). However, there was not a significant difference in the errors of the aMCI and the AD group (χ2(1)=.665; p=.415) and also between the naMCI and CP groups (χ2(1)=.588; p=.446). The difference between the AD and naMCI was shown to be significant (χ2(1)=8.792; p=.003).

An extension to the model that included participant age revealed that the group main effect remained significant (F(3,235)=8.173; p<.0005), whilst age was not (F(1,235)=.167; p=.684). To provide further verification that age was not a confounding factor, an age-matched sample was entered in the analysis. The results, presented in Table 1, were essentially replicated. There was a significant main effect of participant group for antisaccade errors (F(3,207)=9.295; p<.0005). For antisaccade errors, the AD (N=64; mean proportion=26; SD=28; 95% CI=19-32) revealed an increased error rate compared to the CP group (N=69; mean proportion=10; SD=10; 95% CI=7-12; χ2(1)=16.945; p<.0005) and the naMCI group (N=37; mean proportion=13; SD=12; 95% CI=9-16; χ2(1)=7.183; p=.007). Again, critically, the aMCI group (N=39; mean proportion=30; SD=31; 95% CI=20-40) revealed a significantly higher frequency of errors compared to the naMCI group (χ2(1)=8.327; p=0.004) and the CP group (χ2(1)=17.635; p<.0005). The aMCI group did not differ from the AD group (χ2(1)=.258; p=.612 and nor did the naMCI group from the CP group (χ2(1)=.976; p=0.323).

Further, to confirm whether AST error rate was associated with memory decline we performed a series of correlations between AST failure rate and FCSRT free recall score. Overall there was a negative association between AST error rate and FCSRT free recall (r(180)=-.480;p<.0005) which indicates that an increase in AST errors was associated with poorer free recall on the FCSRT task. This association was also observed when considering the participant groups separately: a significant negative association was found between AST error rate and FCSRT free recall within AD patients (r(42)=-.430;p=.004), aMCI (r(39)=-.439;p=.004), and marginally for naMCI (r(44)=-.288;p=.052). The association was not significant in control participants (r(49)=-.166;p=.243). The results indicate that AST inhibitory control is associated with memory decline in patient groups.

To clarify the spatial-temporal characteristics of the extracted signals in the AST, heatmaps of the gaze positions of each participant group are displayed in Figure 2. The heatmaps that the density and length of the “comet” (yellow) tail is shortest for the CP and naMCI groups; and longest for the AD and aMCI groups. This reflects the findings reported above, that the AD and aMCI generated a high proportion of errors in the AST which are uncorrected. Fewer of these uncorrected errors are seen in the traces of the control and naMCI group. In summary, three salient patterns emerge: (1) There were a number of reflexive saccades to the target location (which should have been inhibited) that increased from the level in CP participants, to the naMCI patients, to the aMCI patients, and finally showing the highest level in the AD patients. (2) Similarly, the time cost to correct the mistakes increases from the CP participants, to the naMCI patients, to the aMCI patients, and finally to the AD patients. Given 1500 milliseconds since target onset, the naMCI patients and the CP participants could correct almost all the mistakes, while the AD and aMCI patients have more uncorrected mistakes. (3) The CP participants revealed a higher proportion of correct antisaccades and corrected errors in comparison to the patient groups (AD, aMCI, naMCI).

## DISCUSSION

We can summarise the key findings from this work: (1) These findings demonstrate for the first time, that the AST can discriminate between people with aMCI and naMCI; and (2) The findings replicated the previously reported impairment in inhibitory control of antisaccades in people with dementia due to AD.

These results confirm that the AST is a promising biomarker for dementia. Given that people with MCI are more likely to develop dementia due to AD than cognitively healthy adults, and in particular that people with (aMCI) are at the highest risk of progressing to a full dementia syndrome, this may also offer an additional prognostic tool for predicting which people with a diagnosis of MCI are more likely to progress to dementia due to AD. Our results support previous literature which has also demonstrated that AD [3,5] and MCI [8,9] participants are impaired on the AST. These findings, when taken together, may therefore indicate that people with AD gradually lose the efficient control of attention and develop impairments of both inhibitory control and eye movement error-correction. There is a growing consensus that eye-tracking now provides an important opportunity for clinicians to detect the very early stages of Alzheimer’s disease, during the MCI phase. Several meta-analyses have been conducted all conclude that there is a clear excess of inhibition errors in AD (e.g [19].). These findings are particularly compelling given the coupling of the impairments that have been replicated in these studies. For example, one recent study [20] replicated the increase in antisaccade errors together with a decrease in the frequency of corrected errors. This pattern of inhibitory impairment has not been found in other neuropsychiatric neurodegenerative such as schizophrenia [21] or Parkinson’s disease [5]. There is therefore little doubt that eye-tracking offers a highly reliable, simple, non-invasive assessment and patient-friendly tool for the cognitive assessment of dementia. In line with previous work, Holden et al [22] have shown that aMCI patients also show the increased antisaccade error rates. However, as one recent meta-analysis confirmed, no previous study has yet compared the different subtypes of MCI in a direct comparison of oculomotor function [19]. Here we show that people with aMCI have significant impairment of inhibitory control that is similar to AD; whilst people with naMCI are relatively unimpaired, with a level of inhibitory control that was more similar to the control group.

Our results suggest that inhibitory control of eye movement may be one of the earliest biomarkers of the onset of AD. Inhibitory control deficits appear to be associated with memory decline. There have been relatively few studies of antisaccades in MCI, and, to our knowledge, no longitudinal studies. In future work, the AST could be studied in people with MCI using a prospective longitudinal study design. We hypothesise that those people with increased antisaccade error rates will be more likely to have poorer memories and may be at risk of developing dementia due to AD. Overall, our results support the role of the AST as a useful supplementary tool for the early detection of decline in people with MCI.

Inhibitory error rates in the antisaccade are sensitive to memory impairment, but may even precede it in a patient with dementia [5]. The results obtained from this study demonstrate that eye movements during the AST could be used to automatically classify participants as being at a higher risk of AD. There are potentially a number of practical implications for this observation. With early detection of AD, the potential for commencing effective interventions earlier are increased.

## MATERIALS AND METHODS

### Participants

Participants were men and women between the ages of 55 and 90, with at least 11 years of education and fluent English-speakers. Of these, 68 were people with dementia due to AD, 42 had a diagnosis of aMCI, and 47 had a diagnosis of naMCI. We also included 92 age-matched cognitively healthy people to act as control participants (see Table 2). Control participants were recruited from the local community or were the spouse/partner of the AD or MCI participants. All participants were white British or European. Participants with MCI or dementia due to AD were recruited through local memory clinics in the National Health Service (NHS) and had received a clinical diagnosis following a full assessment with a dementia specialist. All those in the dementia group met clinical criteria for dementia due to AD, as per NINCDS-ADRDA criteria [23]. Those with a diagnosis of MCI met the following criteria [24]: (1) subjective complaints of memory decline (reported by the person themselves or an informant); (2) objective memory or other cognitive impairment (considered when scores on standard cognitive tests were >1.5 SDs below age/education adjusted norms) with or without deficits in other cognitive domains; (3) intact daily-life activities. To subtype the MCI group further into aMCI and naMCI, we applied the [24] criteria (see below). Participants with intact cognition (as assessed by a series of cognitive tasks; see below) were recruited from the local community. Participants were not eligible for the study if they had a previous history of head trauma, stroke, cardiovascular disease, active or past alcohol or substance misuse or dependence, or any physical or mental condition severe enough to interfere with their ability to participate in the study. Those with a global or specific learning disability were also not eligible to participate in the study. All participants had the capacity to consent to participation in the study and signed informed consent. Ethics’ Committee approval was granted by Lancaster University and NHS Health Research Authority.

**Table 2:** Descriptive statistics (SD) of participants including cognitive assessment (MoCA) scores for each group.

	Dementia due to AD	Amnesic mild cognitive impairment	Non-amnesic mild cognitive impairment	Control participants	*p-value*
Age	74 (7.7)	74 (7.4)	69(6.9)	69 (7.2)	<.0005
Sex (% male)	50%	41%	57%	43%	.297 ns
MoCA total score	20 (5.7)	21(4.5)	25 (2.2)	28 (1.8)	<.0005
FCSRT – Free Recall	17.32 (12.0)	18.7 (7.7)	32.3 (4.2)	36.1 (5.7)	<.0005
FCSRT - Total	36.2 (14.8)	45.1 (4.4)	47.4 (1.3)	47.8 (0.8)	<.0005
Digit span total	15.6 (4.1)	16.4 (3.7)	16.7 (4.8)	18.7 (4.5)	<.0005
Spatial span total	11.3 (3.1)	12.6 (3.1)	13.0 (2.6)	14.6 (2.8)	<.0005

MoCA - Montreal Cognitive Assessment [25]; Free cued selective reminding task free recall and total score [26]; digit span and spatial span [27,28].

### Stimuli and tasks

Eye-tracking was assessed using the AST. Cognition was assessed the Montreal Cognitive Assessment (MoCA [25]), the Free and Cued Selective Reminding Test with Immediate Recall (FCSRT-IR [26]), and the digit and spatial span [27,28].

### Apparatus

We used an EyeLink Desktop 1000 eye-tracker (SR Research) with sampling at 500 Hz. Participants sat 55 cm away from the monitor (60 Hz). Their dominant eye was determined using the Miles test [29] and tracked accordingly. Experiment Builder software (SR Research) was used to control the stimulus events during the eye-tracking tasks.

### Antisaccade task (AST)

Each antisaccade trial was preceded by a 1 second instruction screen stating that the participant should look toward the target. A central fixation target central fixation was displayed in white on a black background. This was displayed for one second. There was a 200ms blank interval before the appearance of the saccade distractor. The saccade distractor (in red) was then presented in a random order 4 degrees away from where the fixation target had been either on the left or right side for 2 seconds. Participants were asked to fixate at the central point then generate the saccade to the opposite position of the screen as soon as the distractor appeared. There were a total of 24 antisaccade trials in AST.

### Montreal cognitive assessment (MoCA)

The MoCA is a brief screening tool for Alzheimer’s dementia that includes the domains of attention and concentration, executive functions, memory, language, visuoconstructional skills, conceptual thinking, calculations, and orientation. The test generates a maximum score of 30; a scores of 26 or more is considered normal.

### Free Cued Selective Reminding Task Immediate Recall (FCSRT-IR)

The International Working Group on Alzheimer’s disease [30] recommended the free and cued selective reminding test (FCSRT [26]) to assess memory, as it showed high sensitivity and specificity in the differentiation of dementia due to AD from healthy controls, and aMCI from naMCI (see [12]). Participants were asked to memorise line drawings of a set of easily recognised objects (e.g., grapes) which belonged to unique category cues (e.g., fruit). 16 items to be learned were presented four at a time on a card, one picture in each quadrant. Participants were asked to search each card, then point to and then name aloud each item (e.g., grapes) after its cue (fruit) was verbally presented. The card was then removed, and the participant immediately performed a cued recall of just those four items on the basis of the category cue. Participants were reminded of any items that were not recalled by presenting the cue and the item together (e.g., the vehicle was a train). This procedure was repeated for all 16 pictures across the four cards. Participants were then given a test phase that consisted of three recall trials. Each test was preceded by a 20 second counting distractor task. Each recall trial consisted of two parts; after two minutes of free recall, category cues were provided for items not retrieved during the free recall phase. If subjects failed to retrieve the item when provided with the category cue, they were reminded by the presentation of both the cue and the item together. A measure of free recall and a measure of cued recall was obtained by calculating the correct responses (both out of a total of 48). This task has high validity as it has been used extensively in informing MCI and AD diagnoses [24]. [24] recommends that scores equal to or below 27 on the free recall score indicate aMCI, whilst scores of 28 and over indicate naMCI.

### Digit span

In the digit span task [27,28], which assesses phonological memory function, participants were verbally presented with strings of single digit numbers. Once the string had been presented, the participant was asked to recall the digits in the correct serial order. The number of digits presented gradually increased during the course of the experiment, starting with two and going up to a maximum of nine. Two trials were presented at each level and a participant must get both correct in order to progress to the next level of the task. Participants then completed a reverse digit span task where participants need to reverse the order of the presented sequence. Again, the number of digits presented gradually increased from two to eight. Two trials were presented at each level with both correct needed in order to progress. A total digit span score was then calculated out of 30 by summing the correct trials together.

### Spatial span

In the spatial span task [27,28], participants were presented with an array of nine squares. Squares in the array were selected one at a time by the experimenter. At the end of the sequence, it was the task of the participant to indicate the locations of the selected squares in the correct serial order. The number of squares selected in sequence increased over the course of the experiment from two up to a maximum of nine. Two trials were presented at each level and a participant had to get both correct in order to progress. A reverse version of the task was also performed with the participant indicating the sequence in the backwards order. A measure of total spatial span score was calculated out of a total of 32.

### Data processing

**Figure 3:**
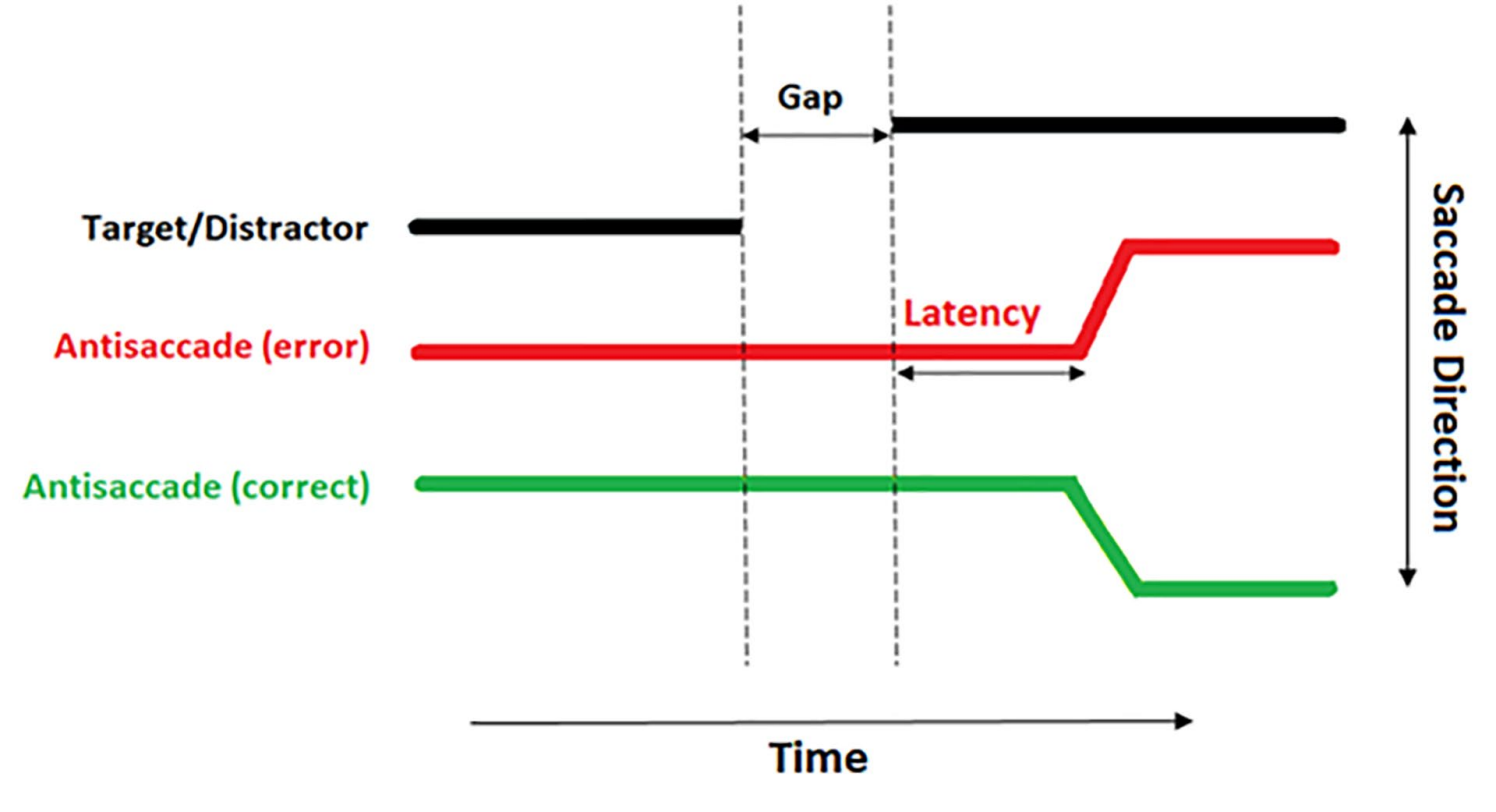
**A diagrammatical representation of an antisaccade trial.** The thick black line demonstrates the location of the distractor. The red line demonstrates the eye incorrectly moving toward the distractor. The green line demonstrates a correct antisaccade away from the distractor.

The raw eye tracking data exported from the EyeLink DataViewer software were analysed offline in bespoke software [31] with the following features: Noise and spikes were filtered by removing all the frames where the velocity signal was greater than 1,500 deg/s or the acceleration signal was greater than 100,000 deg^2^/sec. All the fixations and saccadic events were detected by the EyeLink parser. All the saccades extracted for each trial as well as a range of spatial and temporal properties measured for each saccade were then stored in a table. Microsaccades with amplitude less than 0.7 deg were filtered from the data. The latency of the saccade was measured from the onset of the saccade to the target onset. Only the saccades made within the time window 80-700 ms after target onset (see Figure 3) were included to avoid ‘anticipatory saccades’ i.e. saccades which are initiated prior to the presentation of the distractor.
